# Research progress on surgical approach and intestinal anastomosis methods for laparoscopic right hemicolectomy: A review

**DOI:** 10.1097/MD.0000000000043956

**Published:** 2025-08-22

**Authors:** Jia-wei Wang, Qian Li, Ye Wang, Li-shuai Xu, Song Wang, Cheng-wei Wu, Ting-ting Cao, Ya-bin Xia, Xiao-xu Huang, Li Xu

**Affiliations:** a Department of Gastrointestinal Surgery, The First Affiliated Hospital of Wannan Medical College, Yijishan Hospital of Wannan Medical College, Wuhu, Anhui, China; b Anhui Province Key Laboratory of Non-Coding RNA Basic and Clinical Transformation, Wuhu, Anhui, China.

**Keywords:** access routes, anastomotic approach, laparoscopic right hemicolectomy, right colon cancer, surgery

## Abstract

Patients with tumors located in the cecum, ascending colon, and hepatic flexure of the colon often use laparoscopic right hemicolectomy. Although with the help of laparoscope, the surgery is more visual and refined, due to the different shapes of the blood vessels and the different positions of the frequently occurring blood vessels, the surgeons face challenges during surgery. Improper operation may cause unnecessary damage. Therefore, in order to better solve the problem of variation and reduce intraoperative side injuries, we conducted a literature review on the surgical approach and the guidance of intraoperative anatomy, in order to find more reasonable and simplified surgical methods. At the same time, we conducted a review of various intestinal anastomosis methods to identify more effective approaches, aiming to reduce intestinal tension at the anastomosis site, decrease the incidence of anastomotic leakage, and better promote the recovery of postoperative intestinal function.

## 1. Introduction

The latest global cancer data show that the incidence of colon cancer in China ranks third among malignancies, and this disease is associated with the second highest mortality rate among cancers.^[1]^ Compared with East Asian countries such as Japan, which also have a high prevalence of colon cancer, China is characterized by a low rate of early diagnosis and a high proportion of patients in the progressive stage of colon cancer. Although chemotherapeutic drugs play an important role in the treatment of colon cancer,^[2,3]^ the treatment principle is still a comprehensive treatment based on surgical resection, and laparoscopic right hemicolectomy (LRHC) is considered to be the standard surgical treatment^[4]^; it has the advantages of a shorter hospital stay, less postoperative pain, and fewer complications than traditional open surgery, though both have similar disease-free survival rates after surgery.^[5,6]^ Currently, there are 2 main surgical approaches for radical surgery with right hemicolectomy internationally, and Japanese scholars^[7]^ have proposed the D3 radical principle. This principle emphasizes the importance of D3 lymph node dissection. For progressive right hemicolon cancer, lymph node dissection is recommended to the level of the root of the superior mesenteric artery. In 2009, Hohenberger et al^[8]^ in Germany first proposed the concept of complete mesocolic excision (CME) as a standardized surgical procedure for colon cancer, that is, a sharp separation is performed along the plane of the embryonic fascia that wraps the mesentery of the colon. The advantage of CME is its complete removal of the midcolon and its surrounding lymph nodes as well as the reduced risk of tumor spillage into the peritoneal cavity.^[9]^ Extensive clinical practice has demonstrated that both D3 radical surgery and application of CME principles can provide good oncologic results.^[10,11]^ However, laparoscopic radical right hemicolectomy is difficult due to the high number of vascular variants and the complexity of the anatomical level. Therefore, it is necessary to study the influence of different factors on surgical treatment.

In this paper, we conducted a literature review on surgical access and how to guide intraoperative dissection to find a more rational and simplified surgical approach. At the same time, we reviewed different methods of intestinal anastomosis to explore better intestinal anastomosis and reduce postoperative complications.

## 2. Surgical approaches for LRHC

The main surgical approaches for LRHC include the lateral approach, cephalad approach, medial approach, caudal approach, and combined approach. These different surgical approaches should be used in order to safely and appropriately resect the tumor, depending on the individual’s constitution and tumor pathology. Although the tissue structure is often constant, the surgeon must be alert to structural variations. In addition, we have redrawn a table summarizing surgical access for right hemicolon cancer, what is shown in Table 1.

**Table 1:** The surgical access for right hemicolon cancer.

Operation	Key points of the operation	Advantage	Disadvantage	Development
Lateral approach	Freeing the colon from the outside to the center.	1. Simple and easy to learn. 2. Safe.	1. Excessive stretching of the intestinal canal. 2. Direct contact with the tumor.	Applied to 1. Tight mesenteric vascular adhesions. 2. Unclear tissue level and anatomy.
Cephalolateral approach	Exposure of the junction of the right gastric omental vein and the trunk of Henle.	1. Compliance with the “NO-Touch” principle. 2. Reduction of bleeding.	1. Blood vessel damage. 2. Lymph nodes not completely cleared.	Applied to Higher lymph nodes.
Intermediate approach	The ileocolonic vessels and superior mesenteric vein are used as anatomical landmarks.	1. Minimal invasive. 2. Compliance with the “NO-Touch” principle.	1. Gaps not easily visible. 2. Lymph nodes not completely cleared. 3. Not applicable to obese patients.	Applied to normal weight patients.
Caudal approach	Incision of the colonic mesentery from the right margin of the superior mesenteric vein at its fusion with the posterior peritoneum.	1. Easy to find the correct anatomical plane. 2. Reduced collateral damage.	1. Difficulty in exposing the surgical area. 2. Complications abound.	Applied to 1. Obese patients. 2. Patients with tethered edema.

### 2.1. Lateral approach

**Figure 1.**
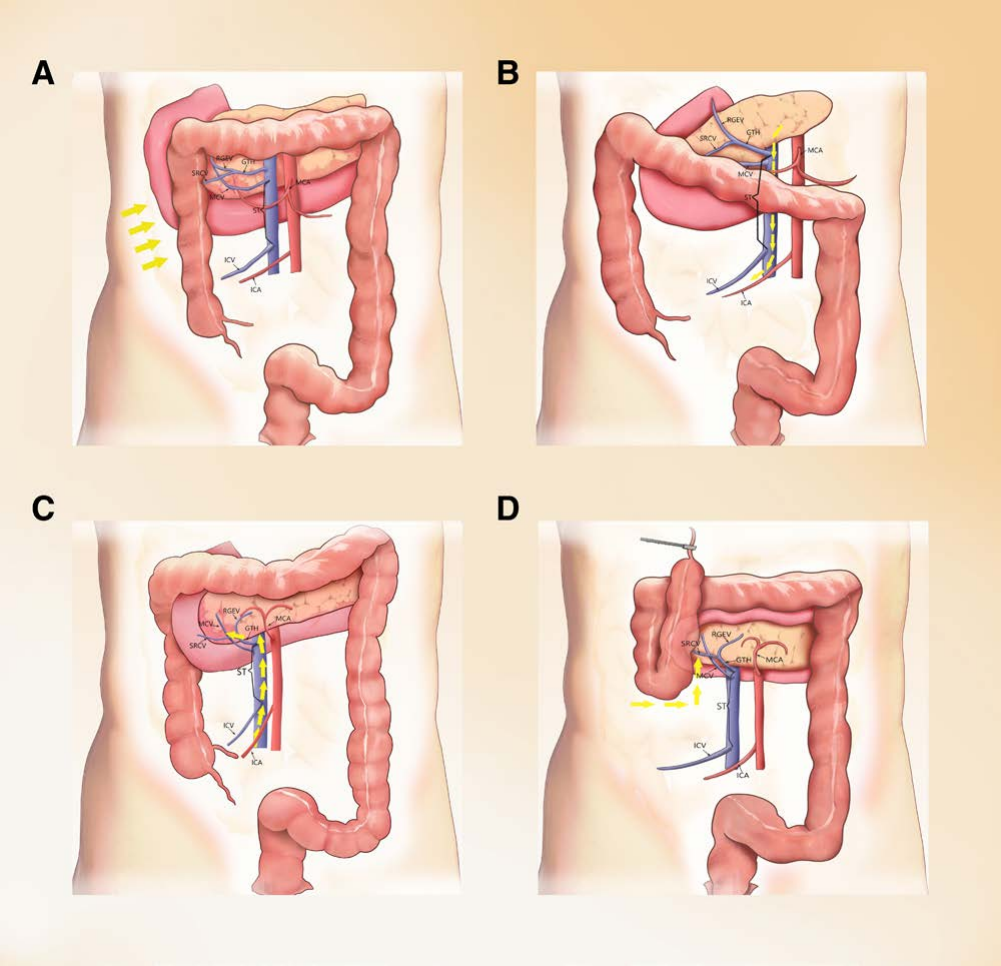
Surgical approaches for LRHC. (A) Lateral approach; (B) cephalolateral approach; (C) intermediate approach; (D) caudal approach. GTH = gastrocolic trunk of Henle; ICA = ileocolic artery; ICV = ileocolic vein; MCA = middle colic artery; MCV = middle colic vein; RGEV = right gastroepiploic vein; SRCV = superior right colic vein; ST = surgical trunk.

The lateral approach is a common surgical approach when traditional open surgery and laparoscopic techniques are just emerging. It is necessary to explore the surgical field when the surgery begins. First, the abdominal cavity is explored for distant metastasis and to determine whether the tumor invades the plasma layer. At the root of the right mesentery, there is a constant line at the fusion of the visceral fascia and the retroperitoneum, which is called Toldt line, along which the right Toldt gap is entered, and the colon and its mesentery are freed from the outside to the inside in turn. Then, the right hemicolonic mesenteric vessels are treated, and finally, the intestinal canal is cut. The lateral approach is mainly suitable for cases with tight mesenteric vascular adhesions and unclear tissue level and anatomy, and its biggest advantage is excellent safety and a short learning curve. However, excessive stretching of the intestinal canal often leads to changes in anatomical landmarks during surgery, resulting in access to the wrong anatomical plane, causing intraoperative bleeding and even damage to retroperitoneal organs. It is also considered incompatible with the principle of radical tumor treatment because of the suspicion of direct contact with the tumor. This approach is shown in Figure 1A.

### 2.2. Cephalolateral approach

Some scholars^[12]^ have proposed that the cephalolateral approach is also an approach recognized by clinical practice and is a technically feasible and safe method for treatment with right hemicolectomy. When performing a cepcephalolateral approach, the gastrocolic ligament is first freed. After freeing the gastrocolic ligament to expose the transverse colonic mesentery, Toldt space is entered via pancreatic inferior border incision. The middle colic vein is ligated, followed by colonic/mesenteric mobilization with vessel ligation. The right colon is then freed along the fused fascial plane prior to bowel transection. The cephalolateral approach is in accordance with the oncology “NO-Touch” principle; second, by freeing and ligating the middle colonic vein, bleeding during the separation of the middle colonic artery can be reduced. It is also simpler and safer for clearing the lymph nodes and reducing vascular damage. However, the cephalic approach is prone to damage the right colonic vein and the gastrocolic trunk, causing uncontrollable hemorrhage. Even the superior mesenteric vein (SMV) and middle colonic artery can be damaged during hemostasis, and it is difficult to completely clear their root lymph nodes. However, the Japanese scholar Matsuda et al^[13]^ pointed out from the perspective of membrane anatomy that the cephalic approach can reverse the torsion and fusion of the transverse colon that occurs during embryonic development and can simplify the complex anatomy of the transverse colon, which is more in line with human embryonic development. This approach is shown in Figure 1B.

### 2.3. Intermediate approach

With the development of laparoscopic techniques, the intermediate approach has gradually been accepted by most scholars and is currently the most widely used approach. Usually, the operation can be performed in an inside-out and bottom-up manner. The middle approach is divided into a complete middle approach and a combined middle approach according to the manner of dissecting the middle colon blood vessel and Henle trunk. In the complete intermediate approach, the ileocolic vessels and SMV are used as anatomical landmarks. After abdominal exploration for distant metastases and to determine whether the tumor has invaded the plasma layer, the mesentery is incised along the lower edge of the ileocolic vessels to enter the right Toldt interval and anterior renal fascia, reveal the duodenum and pancreas, free the right hemicocele, and separate and ligate the vessels. Then the gastrocolic ligament is gradually separated from the small intestinal mesentery and the hepatic colic ligament, and finally, the intestinal canal is cut. The use of an intermediate surgical approach to achieve CME is more in line with the principle of NO-Touch for tumors, providing a perfect combination of minimal invasiveness and tumor-free precaution. At the same time, the operator can use the gastrocolic ligament and lateral peritoneum as anatomical landmarks, which makes it easier to find the correct anatomical plane, reduce intraoperative bleeding and facilitate a smooth operation. On the basis of the complete intermediate approach, He et al^[14]^ proposed a “page-turning” surgical path for CME of right hemicolectomy and summarized a “point, line, and surface” technique. The “point” refers to the anatomical projection point of the ileocolic vessels as the starting point to the opening of the colonic mesentery at its inferior edge; the “line” refers to the main line of the SMV, the dissection and ligation of each branch vessel, and the clearance of regional lymph nodes. The “surface” refers to the right posterior colonic space or the transverse posterior colonic space, both of which are free of avascularity and can be used as natural surgical planes. In the operation, the emphasis is on “from point to line, from line to surface.” This echoes the inside-out and bottom-up characteristics of the intermediate approach and is also more in line with the principle of “treating the vascular root first” in radical tumor treatment. The intermediate approach is considered the classical approach for laparoscopic radical colorectal cancer surgery and can be applied to most colorectal cancer surgeries, including radical right hemicolectomy. It is characterized by prioritizing the treatment of dissected vessels, which makes lymph node dissection simpler and more in line with the “NO-Touch” principle; meanwhile, the surgical trunk along the SMV is dissected from the bottom up to dissect out each arterial branch and the venous geniculate branches emanating along the way. Therefore, there are advantages for the management of vascular variants. However, this approach also has disadvantages. First, the root of the vessels is ligated first, and the gap is not easily revealed completely; thus, the pancreas and vessels are easily injured accidentally during the “climbing” of the lower edge of the pancreas, resulting in bleeding. Second, the peritoneum is incised from the lower edge of the ileum, and lymph nodes in the group 203 area near the origin of the ileocolic vessels cannot be completely cleared, which affects the integrity of lymph node clearance. Third, the SMV of obese patients cannot be well visualized, and there is a risk of entering the wrong level, which necessitates higher requirements for anatomical level recognition and operative field exposure; in turn, longer periods of training and learning are required for the operator and assistant to gradually master this method. This approach is shown in Figure 1C.

### 2.4. Caudal approach

The caudal approach is a surgical approach based on the development of laparoscopic techniques and is generally used in cases of obesity and tethered edema. The caudal approach includes a complete caudal approach and a combined caudal intermediate approach, called the caudodorsal and caudoventral approaches. First, Toldt line is used as the anatomical landmark to find the fused fascial gap between the colonic mesentery and the retroperitoneum. Toldt gap is extended by entering the posterior gap of the right hemicolectomy to expose the pancreatic head and duodenal segments, medial to the left side of the SMV, right to the right edge of the perineal vessels, and superior to the duodenum. The peritoneal projection of the superior mesenteric vessels is used as the starting point, and the SMV is dissected and passed through the right fusion gap; it is separated from the caudal side to the cephalic side, and each colonic vessel is cut off after ligation. Dissection of the gastrocolic ligament is performed to access the small omental sac, and the gastric and colonic mesentery are separated, and the fusion gap is passed through to the caudal gap. Then, the right hemicolectomy vessels are treated, the right hemicolectomy is fully freed, the corresponding lymph nodes are treated, and finally, the intestinal canal is cut. This approach facilitates the operator finding the correct anatomic level and reduces the damage to the retroperitoneal organs and the integrity of the mesentery. Its disadvantages are the difficulty in exposing the operative field, the difficulty in performing the freeing and ligation of the vessels, and the high risk of intraoperative complications such as hemorrhage from the Henle stem.^[15]^ Furthermore, the caudal approach is similar to the lateral approach in that it carries the possibility of intraoperative contact with the tumor. This approach is shown in Figure 1D.

### 2.5. Combined caudal–medial approach

In 2001, Japanese scholars proposed caudal ventral (caudal median approach) in right colon cancer.^[16]^ After first exploring the abdominal cavity for distant metastases and to determine whether the tumor has invaded the plasma layer, the ileocecal region is used as a marker to fully reveal the Toldt line, enter the fused fascial space and expand, revealing the duodenum and head of the pancreas in the upper part, medially to the posterior aspect of the SMV and laterally to the peritoneal reflex of the right paracolic sulcus; the SMV is used as a marker to pass through the posterior gap with the right colon, manage the ileocolic and mesocolic vessels, adjust the position head high and foot low, disconnect the gastrocolic ligament outside the vascular arch of the gastric omentum into the small omental sac, and separate the stomach from the transverse colonic mesentery; the posterior interstitial space of the transverse colon is entered and connected with the anterior interstitial space of the pancreaticoduodenum, which would already be free. The right hemicolectomy is then treated, the right hemicolectomy is fully freed, the corresponding lymph nodes are treated, and finally, the intestinal canal is cut. The combined caudal–medial approach is a new type of approach, and the combined caudal–medial approach combines the advantages of both approaches, not only reflecting the advantages of the clear anatomical level of the caudal approach but also bringing into play the advantages of the intermediate approach in managing blood vessels; this is both more feasible and safer and can greatly improve surgical efficacy.

### 2.6. Combined cephalic–caudal approach

Scholars^[17,18]^ have proposed that both cephalic and caudal approaches are clinically feasible and safe options for laparoscopic radical surgery with right hemicolectomy in colon cancer, but the combined application of these 2 methods is less common, and research on the efficacy has not been clarified. Our scholars Zou et al^[19]^ proposed a combined cephalad–caudal laparoscopic right hemicolectomy approach for radical right colon cancer in 2015. The gastrocolic ligament is freed first, and the transverse colonic mesentery is freed to fully expose its anterior lobe. The mesentery of the transverse colon is dissected from left to right along the lower edge of the pancreas, the anterior page is resected, and the right colonic vein and Henle trunk are exposed. The Toldt gap is then expanded. From the caudal side to the cephalic side, it is extended to the duodenum, pancreas, and mesenteric vessels, left to the left side of the SMV and right to the mesentery of the paracolic sulcus of the colon, and the colonic vessels are ligated. Then, the right hemicolorectal vessels are treated, the right hemicolectum is fully freed, the corresponding lymph nodes are treated, and finally, the intestinal canal is cut. Its advantages include the safe clearance of lymph nodes along the SMV after entering the retroperitoneal space by protecting the ureter, as well as the possibility of a shorter learning curve. Overall, the combined cephalad-caudal approach has advantages in the management of surgical difficulties, improvement of surgical safety and quality control, and teaching promotion and popularization.

Currently, an increasing number of scholars have proposed a modified approach, and Du et al^[20]^ proposed a combined cephalic-medial approach in which the bleeding volume and hospital stay were not significantly different from those of the traditional approach. Zhang et al^[21]^ modified the caudal approach and proposed the tunnel method to complete CME, which is more suitable for beginners, with less blood loss and shorter operative time compared to the traditional medial approach. In contrast, Li et al^[22]^ noted that the postoperative gastrointestinal gas resuscitation time of the combined caudal-medial approach was shorter than that of both the combined cephalic–caudal approach and the conventional approach; the combined cephalic–caudal approach could result in fewer postoperative complications than the combined caudal–medial approach, whereas the conventional approach had a shorter hospital stay. Therefore, different surgical approaches have their own advantages and disadvantages, and the operator can choose the appropriate approach according to his or her own habits and patient conditions. At the same time, the team’s cooperation is also crucial.

The key point of laparoscopic right hemicolectomy is entering Toldt space quickly and accurately. If the correct plane is not entered, the mesenteric vessels may be damaged, resulting in bleeding, or the ureter, duodenum, pancreas, other organs, or even the SMV trunk, may be damaged, resulting in intraoperative hemostasis. Second, when entering Toldt interval, quickly and accurately dissecting the superior mesenteric arterioles and their branches, especially the common gastrocolic trunk, is also important. There are many vascular anatomical variations in this area, which increases the ease of causing hemorrhage that may be difficult to stop. Therefore, searching for a better surgical approach and standardizing physicians’ intraoperative operations are key to preventing injury to the surrounding organs and improving the prognosis.

## 3. Anastomotic approach for LRHC

In addition to the choice of surgical access, this procedure remains controversial in the choice of anastomotic approach. Numerous articles have highlighted the importance and complexity of ileocolic anastomosis,^[23]^ which can be performed by intracorporeal (IA) or extracorporeal anastomosis (EA), lateral-lateral or end-lateral anastomosis, anastomosis or manual suturing.^[24]^ Many authors have tried to standardize the surgical technique and discussed its possibilities. However, we can affirm that the surgical technique has not been standardized.

### 3.1. In vivo anastomosis and ex vivo anastomosis

With the vigorous development of laparoscopic techniques, right hemicolectomy has evolved step by step from open right colectomy, hand-assisted laparoscopic right hemicolectomy, and laparoscopic-assisted right hemicolectomy to complete laparoscopic right hemicolectomy and single-incision laparoscopic right hemicolectomy, and we can easily find that the length and number of surgical incisions are gradually decreasing, the anastomotic location is gradually decreasing, and the anastomosis location is shifting from EA to IA.^[25]^ Laparoscopic right hemicolectomy is the standard surgical approach for the treatment of right-sided colon cancer, and ileal anastomosis can be performed in EA or IA to restore the continuity of the GI tract. Several previous studies have compared the efficacy of IA with EA anastomosis after laparoscopic right hemicolectomy, with most studies concluding that IA is associated with better outcomes, reduced short-term morbidity and shorter hospital stay, and reduced postoperative infections and overall complications, with only a few studies concluding the opposite.^[26,27]^ By retrospectively analyzing the efficacy of 56 patients undergoing total laparoscopic right hemicolectomy with 3-step stapled IA for colon cancer, Tu et al demonstrated a shorter anastomosis time, less intraoperative blood loss, less postoperative pain, and earlier recovery of intestinal function compared with EA.^[28]^ IA is advantageous in terms of time to postoperative bowel ventilation and the option of a suprapubic incision, which increases both aesthetic results and reduces the incidence of postoperative infection.^[29,30]^ While postoperative incisional hernia and intestinal obstruction have been a problem for surgeons, studies have shown that IA can reduce both of these conditions.^[31,32]^ Compared with EA, IA reduces intraoperative side injuries,^[33]^ which may be explained by less intraoperative bleeding, clearer visualization during the procedure, and less mesenteric damage, avoiding distortion of the mesentery, which is common in EA.^[34]^ Interestingly, what is more controversial is the potential fecal infection of the abdominal cavity when performing IA, and scholars have suggested that a suction device or gauze can be used or placed below the intestinal incision margin or that oral antibiotics can be used prophylactically to fight the infection.^[35]^ However, it is obvious that performing IA is extremely difficult and requires a high level of laparoscopic operating expertise, which may increase the duration of the procedure, but the duration of the procedure will gradually decrease as the experience and competence of the operators improve.^[36]^ In 2003, Casciola et al^[37]^ completed several cases of complete laparoscopic right hemicolectomy and intracorporeal ileocolic anastomosis (IIA), which showed that this anastomosis is safe and feasible and has the advantages of less postoperative trauma and shorter hospital stay compared with laparoscopic-assisted right colectomy (LARC).^[38,39]^ However, due to the difficulty of this approach and the longer operative time needed, IIA has not replaced extra-abdominal ileocolic anastomosis as the mainstream. In recent years, with the development of intestinal anastomotic devices, great attention has been given to complete laparoscopic right hemicolectomy as well as IIA. In 2016, Abrisqueta et al stated that IIA is a safe and feasible anastomosis procedure with fewer complications and can avoid some of the disadvantages of EA.^[39]^ A recent study showed mild postoperative pain and early recovery of bowel function after IIA.^[40,41]^ However, an analysis of 195 patients who underwent laparoscopic right hemicolectomy, comparing complete laparoscopic IA with laparoscopic-assisted EA, showed that the former had a significantly higher rate of minor complications, which could be reduced by using experienced colorectal surgeons, and both had similar postoperative indicators and oncologic outcomes.^[42]^

### 3.2. Side-to-side or end-to-side anastomosis

In 1968, Steichen first proposed that 2 types of anastomoses could be used to complete ileocolic anastomosis after radical resection for right hemicolectomy: the first was a functional end-to-side (ES) or side-to-side anastomosis (SS) using a linear cutting closure; the second was an ES using a tubular anastomosis.^[43]^ To complete the extraluminal anastomosis, a right transrectal incision is first made, an incisional protection ring is placed, the intestine is removed from the site of the lesion, and the right hemicolectomy is performed ex vivo. For functional SS, a 1-cm incision is made at the ileum and colon end using an electric knife through which a linear cutter closure is placed to complete the lateral anastomosis between ileum and colon, and then 2 small incisions are closed using a linear cutter closure. For ES, the terminal ileum is cut off, and a tubular anastomosis staple anvil is inserted. The tubular anastomosis is inserted from the distal end of the transverse colon, and the anastomosis mandrel is pierced through the mesentery at 5 cm from the severed end. The mandrel is docked with the staple anvil to close the intestinal canal, and the transverse colon stump is closed with a cutting closure. At the end of the anastomosis, the anastomosis and the intestinal stump are manually reinforced with sutures, and the abdominal incision is closed. A current study has shown that ES is associated with a low incidence of anastomotic leakage.^[44]^ One study showed that ES had a shorter operative time than SS, but in 2021, Min Hyun Kim stated that ES was not functionally superior to SS in terms of recovery criteria after laparoscopic right hemicolectomy, including operative time.^[45,46]^ The possible reason for these 2 contradictory studies is the failure to consider that obese patients will have a longer operative time.^[47]^ Initially, ES and SS were performed ex vivo, and subsequently, with the development of laparoscopic techniques, surgeons focused on techniques that allowed for complete IIA, that is, laparoscopic transection and anastomosis of the intestine and retrieval of the specimen through a small incision or through the natural lumen.^[48]^ The IIA was first proposed by Phillips et al in 1992.^[49]^ Tewari and others^[50]^ first proposed the application of cis-peristaltic lateral anastomosis to ileocolic anastomosis in 2005, which was later introduced in China as the overlapping triangular anastomosis method.^[51]^ Reverse peristaltic lateral anastomosis is also known as functional ES.^[52]^

### 3.3. Mechanical anastomosis or hand suture

**Figure 2.**
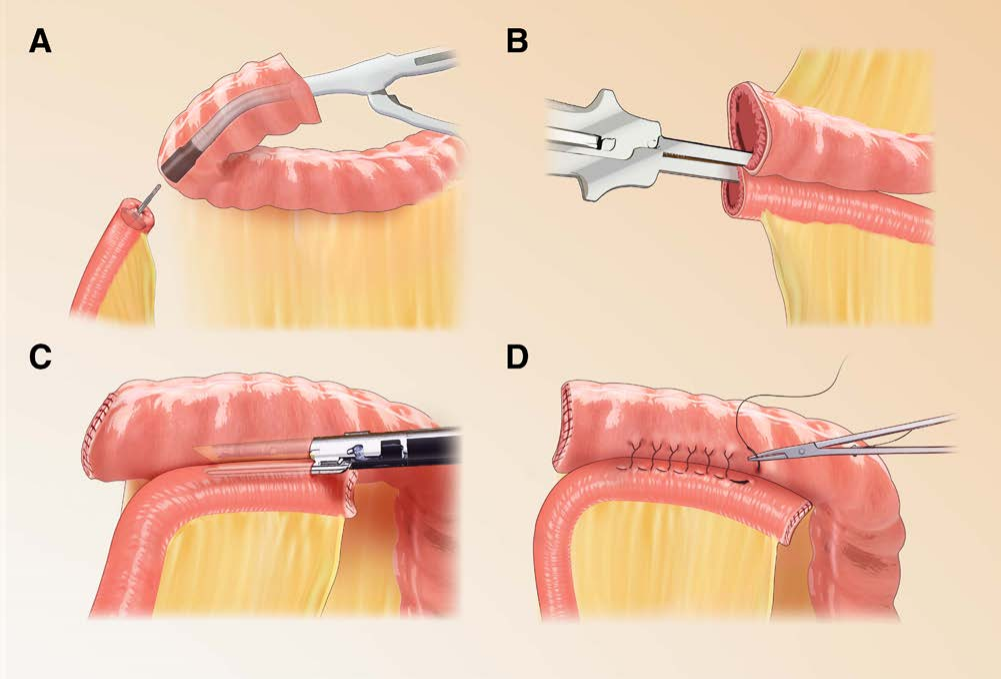
Anastomotic approach for LRHC. (A) End-lateral anastomosis; (B) side-lateral anastomosis; (C) in vivo mechanical anastomosis; (D) in vitro manual anastomosis.

Right hemicolectomy is usually performed using ileocolic anastomosis for gastrointestinal reconstruction and the 2 common methods to construct the anastomosis are mechanical anastomosis or hand suture. There is no consensus on the superiority of mechanical anastomosis over manual suturing for ileocolic anastomosis. The main controversies regarding both include anastomotic leakage rates, bleeding, strictures, and reoperation due to disease recurrence, which would lead to increased morbidity, mortality, length of hospital stay, and hospital costs. In 2011, a Cochrane systematic review including 1125 patients undergoing ileocolic anastomosis found a significant advantage in favor of mechanical anastomosis in terms of anastomotic leakage rates,^[53]^ although subsequent published studies conversely pointed to hand-sutured anastomoses as an independent risk factor for anastomotic leak.^[54]^ A comparative study of laparoscopic right hemicolectomy combined with extracorporeal hand-sewn side-to-side isoperistaltic ileocolic anastomosis and intracorporeal mechanical side-to-side isoperistaltic ileocolic anastomosis was recently reported and showed that intracorporeal mechanical side-to-side isoperistaltic ileocolic anastomosis was more advantageous in terms of operative time and hospital stay^[55]^; indicators such as anastomotic leakage and bleeding showed similar results to those previously reported in the literature.^[56]^ In addition, the cost of hospitalization is also an area of concern. The higher cost of mechanical anastomosis compared to manual suturing is primarily attributable to the expense of specialized stapling devices required for the procedure.^[57,58]^ However, with mechanical anastomosis, the main cost of the procedure can be compensated by a shorter hospital stay. In addition, another cost that may be incurred is the cost of readmission or even secondary surgery due to anastomotic leakage, stenosis, and bleeding. Therefore, the specific cost issues need to be further investigated. The schematic diagram of the anastomosis is shown in Figure 2. At the same time, we created a table to illustrate the advantages and disadvantages of mechanical anastomosis and manual suturing, what is shown in Table 2.

**Table 2:** Comparison of mechanical anastomosis versus manual suturing.

Parameter	Mechanical anastomosis	Manual suturing
Anastomotic leakage	Reviews show significant advantages, recent studies show no significant difference from manual suturing.	Some studies suggest that it is an independent risk factor for anastomotic leakage.
Operative time	Significantly shorter.	Longer duration.
Hospital stay	Significantly reduced.	Relatively longer.
Direct costs	High.	Low.
Cost compensation	Shorter hospitalization may offset device costs.	
Complication risks	Comparable rates of leakage/bleeding/strictures.	Highly controversial leakage risk.
Long-term cost issues	Potentially reduced readmission/secondary surgery costs.	Additional costs from complications.
Technical consensus	No established superiority.	

## 4. Summary and perspectives

At present, the purpose of laparoscopic radical right hemicolectomy is not only to achieve minimal invasiveness but also, more importantly, to complete radical tumor surgery in a standardized manner and improve the survival rate of patients.^[59]^ For the choice of surgical approach for laparoscopic right hemicolectomy, most surgeons perform the procedure using a medial to lateral approach, and Strey et al^[60]^ introduced 8 specific critical views to visualize the anatomy as flipping through “the pages of a book” to complete the procedure. The second most common type of anatomic approach is the bottom-up (combined cephalolateral–caudal approach) approach, first proposed by Zou et al^[19]^ for laparoscopic right hemicolectomy and then by Schulte et al^[61]^ for robotic right hemicolectomy. It can be seen that the development of surgical access has a bright future, but no matter how it develops, the author summarizes the following points that cannot be ignored in the development of laparoscopic right hemicolectomy access: the principle of radical tumors; the principle of CME; the principle of D3 lymph node dissection; reduce the difficulty of surgery and shorten the operation time; and reduce intraoperative bleeding and side injuries and effectively reduce the occurrence of postoperative complications. This shortens the learning curve of the laparoscopic technique, which is easy for surgeons to master and promote. For the choice of anastomosis, there is no standardized surgical technique as yet. However, from the current research data, tubular anastomosis has become a widely accepted technique in laparoscopic surgery,^[62]^ which is the reason for ileocolic anastomosis being favored over end-lateral anastomosis, but in recent years, with the application and development of linear cutting closures, lateral anastomosis has gradually become prevalent.^[63]^ Moreover, in vivo anastomosis and mechanical anastomosis can achieve better surgical results for colorectal surgeons with extensive experience, so it is necessary to increase the learning opportunities for surgeons and train them in surgical skills and corresponding level examinations.

**Figure 3.**
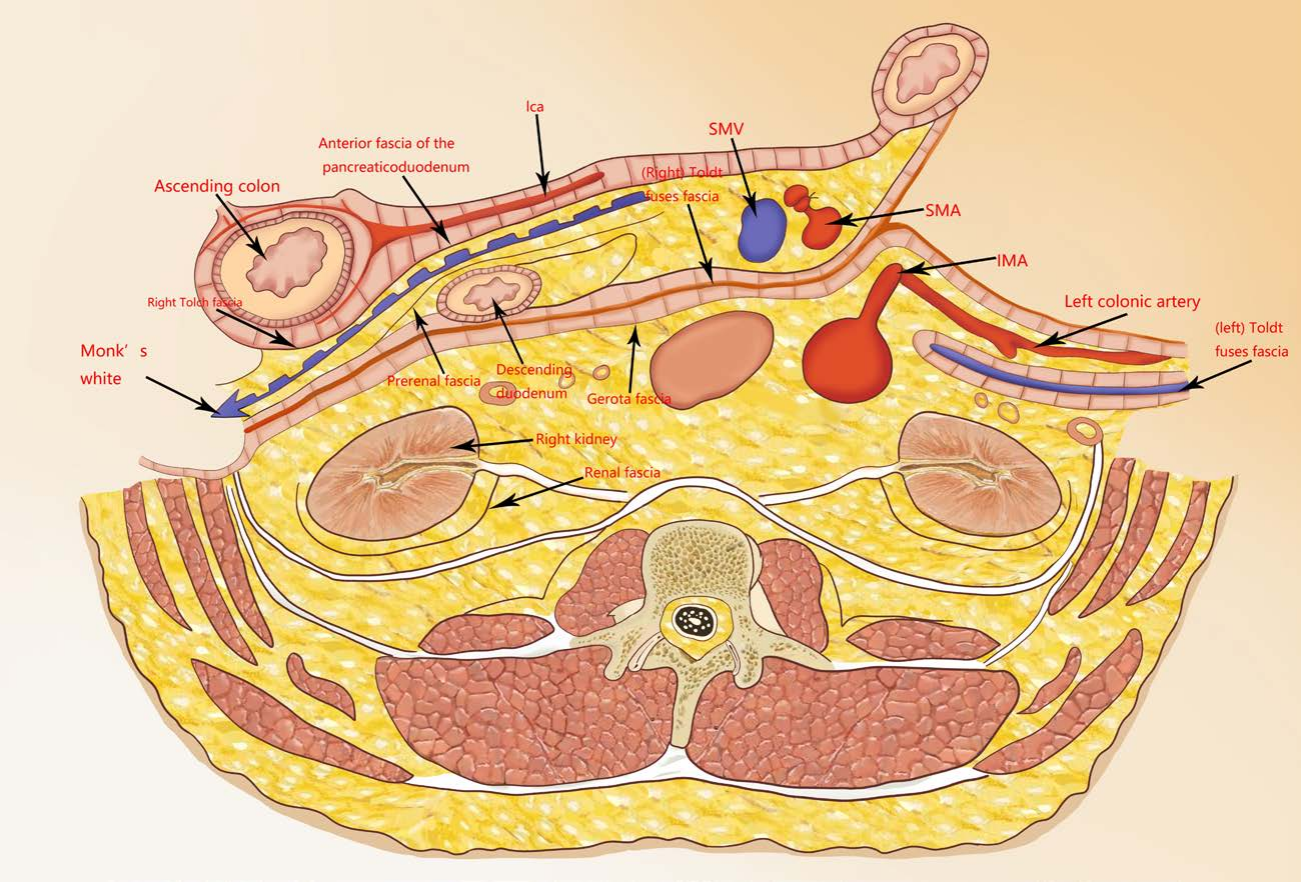
Anatomy of the right hemicolectum. IMA = inferior mesenteric artery; SMA = superior mesenteric artery; SMV = superior mesenteric vein.

The concept of total mesorectal excision, introduced by Heald and others in the early 1980s,^[64]^ has gradually evolved into a curative treatment for rectal cancer. As laparoscopic techniques continued to evolve, researchers sought to extend the advances in the application and survival benefits of total mesorectal excision in rectal cancer to patients with colon cancer, and CME was born; limited evidence now suggests that CME is a more effective strategy for improving specimen quality and survival.^[65,66]^ Triangular anastomosis was initially applied during laparoscopic radical gastric cancer B-I style gastrointestinal anastomosis and gradually became one of the common GI reconstruction modalities for laparoscopic radical gastric cancer surgery.^[67]^ The triangulating stapling method was first reported by Venkatesh et al who used it in colorectal surgery.^[68]^ Currently, with the development of robotics, laparoscopic robot-assisted right hemicolectomy has become a reality. The introduction of robotic systems has technical advantages, including improved visualization, better ergonomics, and precise dissection, further revolutionizing the minimally invasive approach in colorectal surgery.^[69]^ From the current literature, it can be concluded that the application of robotics in right colectomy is feasible and safe for oncological purposes.^[70,71]^ A 2021 META analysis noted that robotic right colectomy was less likely to be converted to open surgery in the context of longer operative times.^[72]^ Whereas the application and development of 3D technology has made colorectal cancer surgery more intuitive and easier, 3D laparoscopic surgery has become a competitive alternative to robotic surgery. 3D laparoscopy allows right colectomy using CME, and robotic surgery should be the preferred approach for complex combined surgery.^[73–75]^ The anatomical structure of the right hemicolectum is shown in Figure 3.

## 5. Conclusion

We reviewed the surgical access and anastomosis for LRHC, whose surgical approach can be comprehensively evaluated based on the results of patients’ imaging examinations, such as CT/MRI and electronic colonoscopy, as well as the results of tumor markers and genetic testing at the time of surgery in patients with right hemicolonic cancer. It is recommended that the principle of tumor radicality be adopted in radical surgery for right hemicolonic cancer to ensure the quality of surgery and enable patients to obtain more satisfactory oncologic outcomes. The choice of surgical access and anastomosis for LRHC seems to remain inconclusive, despite the availability of several methods to predict and eliminate the influence of potential surgical factors preoperatively and postoperatively. In order to improve the prognosis of right hemicolon cancer surgery and reduce the occurrence of postoperative complications, the correct choice of surgical access, and anastomosis should not be neglected, which requires further research and analysis, and the innovation of science and technology is also essential.

## Author contributions

**Conceptualization:** Jia-wei Wang, Qian Li, Ye Wang, Li-shuai Xu.

**Data curation:** Song Wang.

**Formal analysis:** Cheng-wei Wu.

**Funding acquisition:** Xiao-xu Huang.

**Investigation:** Ting-ting Cao.

**Methodology:** Jia-wei Wang.

**Project administration:** Jia-wei Wang.

**Resources:** Li Xu.

**Software:** Qian Li.

**Supervision:** Ye Wang.

**Validation:** Ya-bin Xia.

**Visualization:** Li-shuai Xu.

**Writing – original draft:** Jia-wei Wang.

**Writing – review & editing:** Li Xu.
